# Rapid and Highly Sensitive Detection of C-Reaction Protein Using Robust Self-Compensated Guided-Mode Resonance BioSensing System for Point-of-Care Applications

**DOI:** 10.3390/bios11120523

**Published:** 2021-12-20

**Authors:** Chu-Tung Yeh, Devesh Barshilia, Chia-Jui Hsieh, Hsun-Yuan Li, Wen-Hsin Hsieh, Guo-En Chang

**Affiliations:** Department of Mechanical Engineering, Advanced Institute of Manufacturing with High-Tech Innovations (AIM-HI), National Chung Cheng University, Minxiong Township 62102, Taiwan; tonnyyeh345@gmail.com (C.-T.Y.); d07420005@ccu.edu.tw (D.B.); g09420008@ccu.edu.tw (C.-J.H.); leonli308@outlook.com (H.-Y.L.); imewhh@ccu.edu.tw (W.-H.H.)

**Keywords:** gratings, biomaterials, biological sensing, optical sensing

## Abstract

The rapid and sensitive detection of human C-reactive protein (CRP) in a point-of-care (POC) may be conducive to the early diagnosis of various diseases. Biosensors have emerged as a new technology for rapid and accurate detection of CRP for POC applications. Here, we propose a rapid and highly stable guided-mode resonance (GMR) optofluidic biosensing system based on intensity detection with self-compensation, which substantially reduces the instability caused by environmental factors for a long detection time. In addition, a low-cost LED serving as the light source and a photodetector are used for intensity detection and real-time biosensing, and the system compactness facilitates POC applications. Self-compensation relies on a polarizing beam splitter to separate the transverse-magnetic-polarized light and transverse-electric-polarized light from the light source. The transverse-electric-polarized light is used as a background signal for compensating noise, while the transverse-magnetic-polarized light is used as the light source for the GMR biosensor. After compensation, noise is drastically reduced, and both the stability and performance of the system are enhanced over a long period. Refractive index experiments revealed a resolution improvement by 181% when using the proposed system with compensation. In addition, the system was successfully applied to CRP detection, and an outstanding limit of detection of 1.95 × 10^−8^ g/mL was achieved, validating the proposed measurement system for biochemical reaction detection. The proposed GMR biosensing sensing system can provide a low-cost, compact, rapid, sensitive, and highly stable solution for a variety of point-of-care applications.

## 1. Introduction

Human C-reactive protein (CRP) is an acute-phase-related protein consisting of five monomeric subunits produced in the liver. It is one of the most valuable proteins that can be used as a biomarker of acute inflammation in response to cardiovascular disease. In fact, the risks of myocardial infarction and stroke are strongly correlated with the CRP concentration [1]. Clinically, the median concentration of CRP is 0.8 mg/L and always less than 12 mg/L in healthy people, with higher values in elderly people. For cardiovascular events, the reported cutoff level is 3–4 µg/mL [2]. In addition, the chance of developing cardiovascular disease has been quantified into three levels: high risk above 3.0 mg/L, average risk between 1.0 and 3.0 mg/L, and low risk below 1.0 mg/L [3]. Many applications provide reasonable biomolecular recognitions and their efficiency have been illustrated with different kind of biosensors inclusive of SPR, immunoassay, scanning tunneling microscopy, high resolution spectroscopy, microarrays, and electrochemical sensors [4,5]. Moreover, CRP is not bound to any particular condition, but it can serve as a biomarker to track disease progression and treatment progression for cases such as inflammations, surgeries, burns, cardiovascular diseases, cancers, and tissue necroses. Hence, CRP is a disease-sensitive biomarker in the human body with a more accurate response during the acute phase of a health condition. To adequately detect CRP, in addition to conventional detection techniques such as ELISA, a biosensor with a limit of detection (LOD) below the cutoff level is highly desired [2].

Optofluidic biosensors are miniaturized devices that integrate microfluidics and optics into a single chip. They have enabled biochemical detection with outstanding characteristics including fast operation, low cost, simultaneous quantification, and minimum reagent requirement for a wide range of applications in biomedical research, chemical analysis, clinical research, food safety, and environmental monitoring [6,7]. In addition, optofluidic biosensors provide remarkable performance owing to their high sensitivity, simple optical readout, compact design, robustness against electromagnetic-wave interference, and low complexity [7]. There are two main types of optofluidic biosensors: (1) optical transducers that simultaneously quantify optical signal changes (e.g., phase, amplitude, frequency) in both concentration and refractive index (RI) of chemicals or biological molecules which occupies surface area upon immobilization which depends on shape and size of molecule, and (2) readout devices for sensitive detection of optical signals [7,8,9]. 

Various biosensors have been developed with different measurement principles, including surface plasmon resonance biosensors [10,11,12,13,14,15,16,17], optical resonators [18,19,20], prisms [21,22,23], photonic crystal biosensors [24,25,26,27,28,29,30,31,32], interferometers [33,34,35], fiber-optic biosensors [36,37,38,39,40,41], simple and stable guided-mode resonance (GMR) biosensors having higher sensitivity and improved figure of merit (FOM), [7,42,43,44,45,46,47,48,49,50,51,52,53,54,55,56,57,58,59], GMR sensors based on a combination of optical and electrical techniques [60] and waveguide RI sensors [61]. GMR biosensors are becoming prevalent for chemical analysis and biomolecular detection given their simple structure and high sensitivity [7]. Optofluidic GMR biosensors comprise waveguide layers with 1D periodic structures that can support guided modes by using evanescent waves that extend around the sensing region. At resonance, the incident light beam from far-field can be coupled into the waveguide layer. GMR biosensors based on refractometry have a strong wavelength dependence on the RI of the analyte and on the surrounding medium. As the target molecules are bound to the surface of the waveguide, the RI at the surface of the waveguide is perturbed, resulting in a shift in the resonance wavelength. This shift enables sensitive label-free detection of small changes in RI. The performance of wavelength-resolution-based GMR optofluidic biosensors is mainly characterized by their sensitivity, which is defined by the change in the GMR wavelength (Δ*λ*_R_) with respect to the change in the target RI (Δ*n*_a_). Although high sensitivity has been achieved in GMR biosensors, major challenges remain to be overcome for the practical application of GMR biosensors. The most relevant challenge is the requirement of bulky and expensive instruments such as wavelength-tunable light sources, high-resolution spectrometers, and high-precision angularly resolved rotation stages to capture small GMR wavelength variations produced by RI changes on the structure surface, making it difficult for point-of-care (POC) applications. In addition, time-consuming and complex data postprocessing is required to accurately determine the GMR wavelengths, preventing rapid detection with high throughput.

Instead of focusing on wavelength-resolved GMR biosensing systems, a few studies have been devoted to the design of compact and cost-effective intensity-detection-based GMR biosensing systems [7,26,49,56]. However, the performance of these biosensors remains unsatisfactory. In fact, achieving high-sensitivity biosensing in intensity-detection-based optofluidic GMR biosensors requires improving the LOD. In addition, as practical intensity-detection-based biosensing of biomarkers usually takes tens of minutes, environmental factors may increase noise and undermine the LOD and detection accuracy, especially at low analyte concentrations. Thus, a robust and highly-stable GMR biosensor must be developed for practical applications.

Here we propose and develop a highly stable self-compensated intensity-detection-based GMR optofluidic biosensing system for CRP detection aimed at early and rapid POC disease diagnosis. The biosensor chips were fabricated using special injection-molded techniques to achieve a simple structure and cost effectiveness for mass production. The readout system employs a highly stable white light-emitting diode (LED) as the light source with a bandpass filter to reduce intensity variations caused by environmental factors, such as voltage instability and temperature, and a photodetector (PD) as the optical receiver for real-time detection. In addition, we introduce self-compensation by using a polarizing beam splitter that separates the transverse-magnetic (TM)-polarized light and the transverse-electric (TE)-polarized light from the LED source. The TE-polarized light is used as background signal, and the TM-polarized light is used as the light source for the GMR biosensor. The system exhibits significantly-enhanced biosensing performance in terms of system noise and long-term stability with self-compensation techniques. These results demonstrate a highly stable GMR biosensing system for a wide range of practical point-of-care biosensing applications, food safety, environmental monitoring, and chemical sensing.

## 2. Materials and Methods

### 2.1. Detection System Design

**Figure 1:**
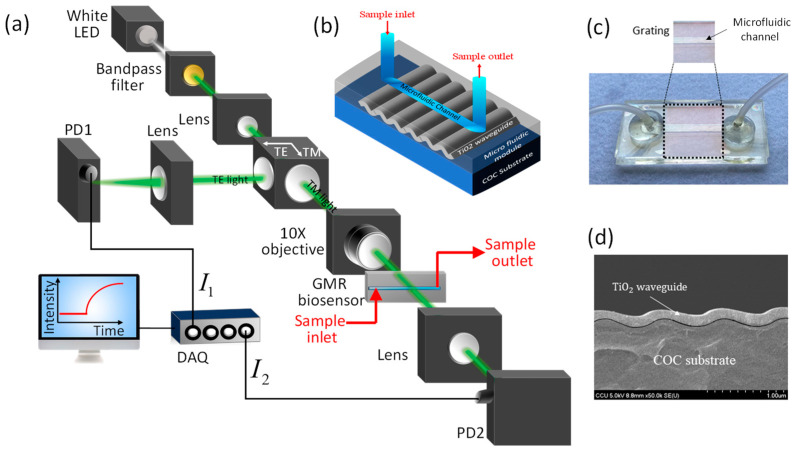
Our proposed GMR biosensing system. (**a**) Schematics of our proposed self-compensated, intensity-detection-based GMR biosensing system. (**b**) 3-D Schematic view and (**c**) optical image of our injection-molded GMR biosensors. (**d**) Scanning electron microscopy (SEM) image of the grating structure with a TiO_2_ waveguide layer.

Figure 1a shows a schematic diagram of the proposed self-compensated, highly stable, intensity-detection-based GMR biosensing system. A cost-effective and stable commercial LED powered by 1 kHz square-wave voltage is used as the light source. The emitted light is filtered using a 500–550 nm bandpass filter and then collimated by a plano-convex lens. A linear polarizing beam splitter is introduced into the system to separate the TM-polarized light and TE-polarized light. Compared with the TE-polarized light, the TM-polarized light induces larger the optical response of the GMR biosensor to changes in the RI [44]. We reshape the TM-polarized light by an adjustable iris, and it is obliquely incident on the substrate of the GMR biosensor via a 10× objective. Hence, the spot size arriving at the GMR biosensor is smaller than the sensing area (i.e., width of microfluidic channel). The GMR biosensor is mounted on a rotation stage to adjust the incident angle of the light beam to shift the GMR wavelength to the spectral range of the light source. The transmitted light is collected using a Si PD via a convex lens. On the other hand, the TE-polarized light is collected by another Si PD via a convex lens. The TE- and TM-mode signals are acquired and converted into photocurrents by the Si photodetectors. These signals are further amplified using in-house current amplifiers with bandpass filters and then converted into digital signals by analog-to-digital converters in real-time. The resulting signals are demodulated in a computer using a digital lock-in amplifier program.

We fabricate cost-effective GMR biosensor chips by combining sputtering and injection molding [7]. A schematic diagram and an optical image of the fabricated GMR biosensors are shown in Figure 1b,c, respectively. The GMR biosensor chips comprise a low-RI cyclic olefin copolymer (COC) substrate (*n* ≈ 1.53, *λ* = 532 nm) with a 1D periodic grating structure with a period *Λ* = 416 nm, and an amplitude *A* = 100 nm. On top of the substrate, a 125-nm-thick high-RI TiO_2_ waveguide layer (*n* ≈ 2.45, *λ* = 532 nm) is deposited using a sputtering reactor with precise step coverage, as revealed by the scanning electron microscopy image shown in Figure 1d. The biosensors are completed by integrating an injection-molded COC microfluidic module with a microfluidic channel of 32 × 3 × 0.2 mm (length × width × height) and two flexible tubes for handling sample solutions. The GMR biosensor chip is affordable (<1 USD per chip), has excellent run-to-run and chip-to-chip stability (standard deviation less than 2.6%) [56], and can be produced with a high throughput.

### 2.2. Working Principle

The sensing mechanism of the proposed GMR biosensor is based on intensity detection using a spectrum-limited LED and a PD [7]. The modulation of the light-intensity signal is identified when analytes with different RIs are injected into the chip, reflecting the sensitivity and resolution of the system and providing real-time detection. However, the sensing performance can be notably affected by instability in the LED light source and environmental temperature variations, thus altering the LOD. When a bioreaction occurs, molecules are attached to the surface of the biosensors by diffusion, which usually requires tens of minutes to reach a steady state. Thus, long-term stability is crucial during the bioreaction for the accurate determination of the analyte concentration, and noise suppression must be performed to enhance bio-sensing.

For compensation in the intensity-detection-based GMR biosensor, the intensity of the light source is tracked to improve stability, as illustrated in Figure 1a. The polarizing beam splitter separates the light beam from the LED source into the TE- and TM-polarized components. The TE-polarized light is read by photodetector PD2 (*I*_2_) to track intensity variations of the LED for compensation, while the TM-polarized light is obliquely incident on the GMR biosensor filled with an analyte having an RI of n. The transmitted light intensity through the GMR biosensor (*I*_1_) is collected using photodetector PD1. The electrical signals from the PDs (*I*_1_ and *I*_2_) are then processed to reduce the noise of the LED light source, thereby enhancing the LOD and stabilizing the system against environmental temperature variations. However, the different optical paths make the orders of magnitudes of *I*_1_ and *I*_2_ differ. Hence, adequate techniques should be devised to compensate for the signal and suppress noise.

### 2.3. Compensation Techniques

To reduce the system noise and enhance the LOD, we designed and experimentally evaluated the three compensation techniques using TM- and TE-polarized signals detailed below.

#### 2.3.1. Direct Signal-Difference Compensation

Direct signal-difference (DSD) compensation uses the intensity received by the two PDs (*I*_1_ and *I*_2_) to directly perform compensation as follows:

(1) $$I_{c}=\left|I_{1}-I_{2}\right|$$

This compensation technique eliminates the drift of the signal in the TM mode to suppress noise from the measured signal.

#### 2.3.2. Weighted Signal Magnification Compensation

Weighted signal magnification (WSM) compensation extends direct compensation. As the magnitudes of the initial signal intensities for *I*_1_ and *I*_2_ are different owing to the differences in the optical paths and optical losses, direct compensation may not suppress noise. Therefore, a magnification relation between *I*_1_ and *I*_2_ should be determined to then adjust the signal strength of the TM and TE modes with the measured magnification.

The measurement for weighted signal magnification compensation can be performed in two steps.

(1). Compare the average absolute values of *I*_1_ and *I*_2_ of the sample solution to obtain compensation coefficient $R_{\mathrm{WSM}}$:

(2) $$R_{\mathrm{WSM}}=\frac{\left|{\bar{I}}_{1}\right|}{\left|{\bar{I}}_{2}\right|}$$

where ${\bar{I}}_{1}$ and ${\bar{I}}_{2}$ are the time-averaged intensities measured from blank solutions. This step allows to compensate for the optical loss of the optical path in TE-polarized light.

(2). Multiply the TE-polarized light intensity by the compensation coefficient and subtract the TM-polarized light intensity to obtain the compensated light intensity signal:

(3) $$I_{c}=\left|R_{\mathrm{WSM}}\times I_{1}-I_{2}\right|\;$$

#### 2.3.3. Weighted Difference Dual-Mode Amplitude Magnification Compensation

Following the abovementioned compensation techniques, a novel compensation method (weighted difference dual-mode amplitude magnification (WDDAM) compensation) is introduced based on the signal amplitude. This technique aims to improve compensation coefficient *R_c_* by calculating the standard derivation of the TM- and TE-polarized light amplitudes and performing a comparison as follows:

(4) $$R_{\mathrm{WDDMA}}=\frac{\sqrt{\frac{1}{N}\sum_{n=1}^{N}{\left[I_{1}^{n}-{\bar{I}}_{1}^{}\right]}^{2}}}{\sqrt{\frac{1}{N}\sum_{n=1}^{N}{\left[I_{2}^{n}-{\bar{I}}_{2}^{}\right]}^{2}}}$$

The compensated signal can then be obtained by:

(5) $$I_{c}=\left|R_{\mathrm{WDDMA}}\times I_{1}-I_{2}\right|$$

### 2.4. Calculation of RI Resolution

We experimentally characterized the sensing performance regarding the RI of the optofluidic GMR biosensors. By varying the concentration of sucrose in analytes, solutions with different RIs n ranging from 1.333 to 1.373 were prepared. The RI experiments started with the injection of a blank deionized water solution with n = 1.333 into the biochip followed by injection of the sucrose solutions with different RIs and a final injection of deionized water. The data acquisition system was used in the synchronous mode to simultaneously record the transmitted light intensity and reflected light intensity, which was compensated by using the developed techniques.

The normalized sensitivity (*S_n_*) and sensor RI resolution (*R_s_*), which represent the minimum detectable change in the RI of the solution, are respectively given by [6]

(6) $$S_{n}=\frac{m}{I_{0}^{avg}}$$

(7) $$R_{s}=\frac{\sigma }{S_{n}}$$

where σ represents the system noise given by the standard deviation of the detected light intensity measured from deionized water, m is the slope of the line relating average transmitted light intensity and RI of the solution, and $I_{0}^{avg}$ is the average compensated light intensity measured from deionized water.

### 2.5. CRP Immunoassay

**Figure 2:**
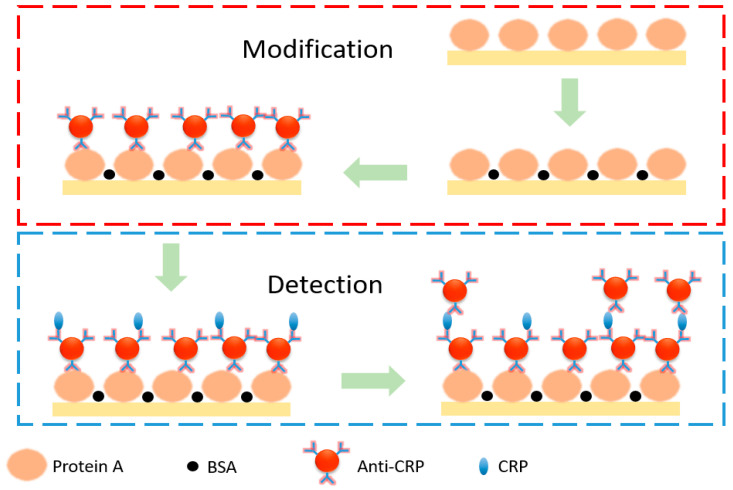
Schematic diagram for CRP modification and detection.

Figure 2 shows a schematic view of CRP modification and detection. We started a biomarker detection experiment with the injection of protein A into the GMR biosensor chip using a syringe pump. After 1 h, bovine serum albumin was injected into the chip. The intensity of the detected light stabilized in approximately 30 min at room temperature. The bovine serum albumin was used to fill void areas that were not modified by protein A on the chip and thus prevent subsequent anti-CRP modification from reacting with the chip instead of protein A. Then, anti-CRP of concentration 5 × 10^−5^ gm/mL was injected to form a capture layer into the chip. After 1 h, phosphate-buffered saline solution was injected into the chip, and after reaching the steady state, the light intensity was recorded for 30 min as blank signals to obtain the average intensity (${\bar{I}}_{0}$) and system noise (σ). CRP of selected concentrations ranging from 5 × 10^−6^ gm/mL to 3 × 10^−7^ gm/mL was then injected into the GMR biosensor, and the light intensity was recorded for 1 h followed by injection of phosphate-buffered saline solution. Then, anti-CRP of concentration 3 × 10^−5^ gm/mL was injected to form an extraction layer into the chip, and the light intensity was recorded to obtain the system response from the average intensity in steady state (${\bar{I}}_{c}$). The process was completed by injecting phosphate-buffered saline solution.

The LOD of the GMR optofluidic biosensor was determined from the real-time optical response. The LOD is defined as the system normalized response (${\bar{I}}_{c}/{\bar{I}}_{0}$) that yields a signal-to-noise ratio of 3 for the system. 

## 3. Results

### 3.1. Refractive-Index Sensing Performance without and with Compensation

**Figure 3:**
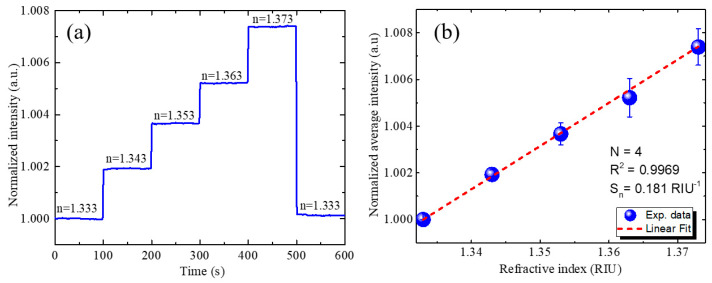
RI sensing results without compensation. (**a**) Real-time responses of optofluidic GMR biosensing system for solutions with different RIs showing variation in intensity. (**b**) Calibration curves for normalized intensity according to RI of sample solution. Four experiments were conducted for the determination of the mean value and the standard deviation depicted as the error bars.

An RI sensing experiment was conducted to evaluate the sensing performance. Figure 3a shows the real-time RI sensing results of the GMR biosensing system without compensation. As the RI of the analyte increases, the intensity of the TM-polarized light is also expected to increase. This change in intensity is attributed to the shift in the GMR resonance wavelength caused by the RI change, which modifies the overlap between the LED spectrum and transmittance spectrum [5]. The normalized average intensities according to the RI of the analyte solution were measured, obtaining the results shown in Figure 3b. A low system noise of $\sigma =1.56\times 10^{-5}$ was obtained, and this value is considerably superior to the typical value of $\sigma =1\times 10^{-4}$ of intensity-detection-based GMR biosensing with a highly-stable laser light source [53]. Normalized sensitivity *S_n_* of the system of 0.181 RIU^−1^ was obtained by linear fitting of the experimental measurements. The RI resolution was then determined to be 8.63 × 10^−5^ RIU.

**Figure 4:**
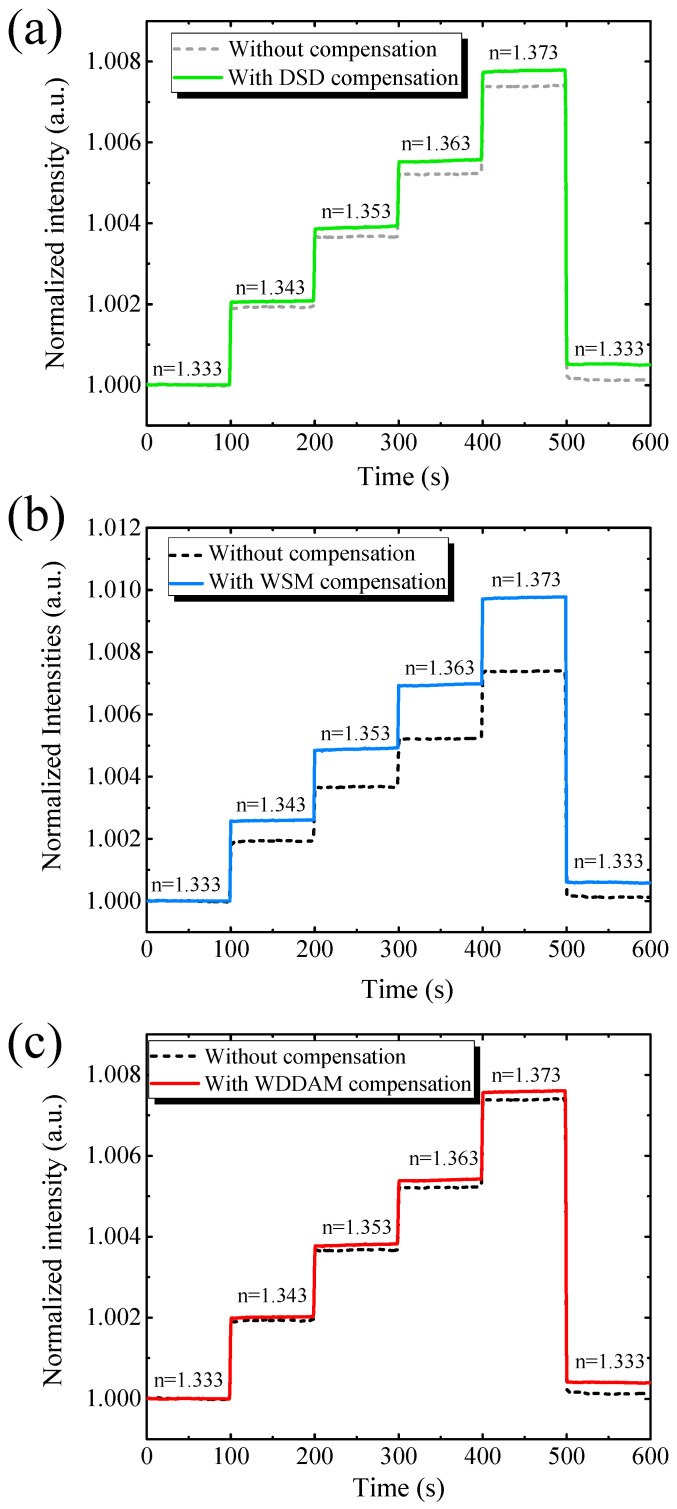
Real-time RI sensing results of optofluidic GMR biosensing system without and with (**a**) direct signal-difference (DSD) compensation, (**b**) weighted signal magnification (WSM) compensation, and (**c**) weighted difference dual-mode amplitude magnification (WDDAM) compensation.

We evaluated the performance of the proposed compensation techniques for RI sensing. Figure 4a shows the RI sensing results with and without direct signal-difference compensation. The responses showed similar trends. The transmitted light intensity increased with the RI, while noise reduced from $\sigma =1.56\times 10^{-5}$ to $\sigma =9.90\times 10^{-6}$, representing an improvement of 36.4% when using the direct compensation. In addition, sensitivity slightly improved from 0.181 to 0.190 RIU^−1^, obtaining a sensor resolution of 5.21 × 10^−5^ RIU over a wide range of 0.04 RIU.

Figure 4b shows the RI sensing results with and without weighted signal magnification compensation. Again, the intensity of the transmitted light increased with the RI. Using this compensation technique, the system noise improved by 44.22%, from $\sigma =1.56\times 10^{-5}$ to $\sigma =8.96\times 10^{-6}$. The results indicate that it is essential to consider the magnitude of the TE- and TM-polarized light signals to mitigate noise. With weighted signal magnification compensation, the sensitivity increased from $S_{n}=0.181$ to $S_{n}=0.239$. Therefore, the sensor resolution was enhanced by 57.8% (*R_s_* = 3.64 × 10^−5^ RIU) compared with no compensation. The suppression of system noise demonstrates the feasibility of the compensation techniques.

Figure 4c shows the RI sensing results with and without weighted difference dual-mode amplitude magnification compensation (WDDAM) compensation. The intensity of the transmitted light increased with increasing RI, and this compensation technique was more effective in suppressing signal noise than the other two techniques. Specifically, the system noise improved by 63.4%, from $\sigma =1.56\times 10^{-5}$ to $\sigma =5.69\times 10^{-6}$, and the sensor resolution improved by 64.5%, from *R_s_* = 8.62 × 10^−5^ RIU to *R_s_* = 3.07 × 10^−5^ RIU.

Table 1 lists the RI sensing performance without and with compensation. For the same measurement settings, the compensation techniques provided different results. The direct compensation ignored the difference in the order of the magnitudes of the TE and TM modes, and different light intensities led to a limited improvement after compensation. For weighted signal magnification compensation, the performance of the system increased, but WDDAM compensation provided the best noise mitigation. Therefore, WDDAM compensation was the most appropriate technique among the evaluated ones for the proposed biosensing system.

### 3.2. CRP Detection

**Figure 5:**
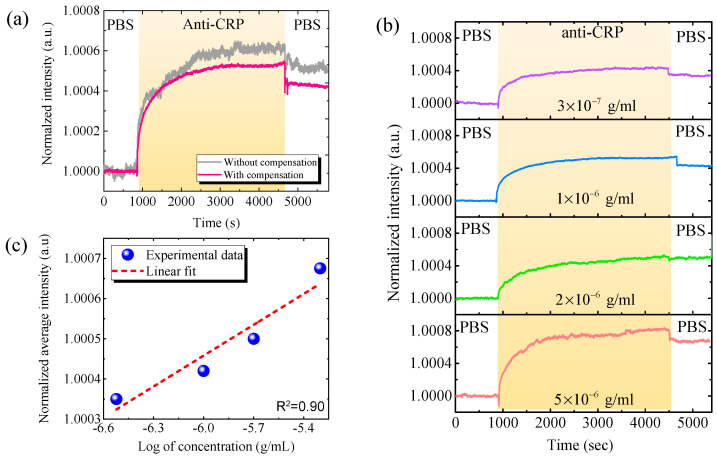
Real-time CRP detection results. (**a**) Real-time responses of optofluidic GMR biosensing system for CRP biomarker (3 × 10^−7^ g/mL) with and without compensation. (**b**) Real-time responses with WDDAM compensation for various CRP concentrations. (**c**) Calibration curves for CRP detection with WDDAM compensation. (PBS, phosphate-buffered saline solution).

We then used the effective WDDAM compensation for CRP detection. Figure 5 shows the real-time detection curve of the CRP biomarker using WDDAM self-compensation. Without compensation, the intensity exhibited slight variations, yielding a system noise of $\sigma =1.19\times 10^{-5}$. With the injection of anti-CRP, the signal increased and then saturated. This behavior is a typical kinetic reaction evident for the capture of anti-CRP by CRP modified on the waveguide surface. If we define the detection time as that required to reach 90% of saturation, the detection time for the anti-CRP/CRP reaction was approximately 20 min using the proposed biosensing system. However, the detection signal varied likely due to system instability and environmental temperature variation over that long detection time. On the other hand, using WDDAM compensation for measurement of phosphate-buffered saline solution, the system noise substantially reduced to $\sigma =2.22\times 10^{-6}$, indicating an improvement by approximately one order of magnitude compared with no compensation. In addition, the signal increased as the anti-CRP was injected, showing the typical kinetic reaction behavior but with much smaller noise, even over the long detection time of 60 min, highlighting the importance of noise suppression for bio-sensing. Figure 5b shows the real-time responses of the GMR biosensing system with WDDAM compensation for various CRP concentrations. A clear bioreaction behavior was observed down to a CRP concentration of 3 × 10^−7^ g/mL. Figure 5c shows the normalized calibration line for different concentrations of CRP biochemical detection. Fitting the data yielded an LOD 1.95 × 10^−8^ g/mL and coefficient of determination *R*^2^ = 0.90. This LOD is lower than the cutoff value of 40–200 μg/mL for accurate CRP detection to diagnose sepsis, demonstrating the effectiveness of the proposed GMR biosensing system for this application. In addition, the experimental results demonstrate the contribution of self-compensation in GMR biosensing to achieve highly stable CRP detection with a low LOD. The LOD may be further enhanced by enhancing the sensitivity of the GMR biosensors in the system.

Several biosensor technologies have been developed for CRP detection. Table 2 lists the analytical performance for CRP detection of the proposed self-compensated GMR biosensor and similar state-of-the-art biosensors. Some biosensors can achieve a low LOD of ~10 ng/mL or better, but their detection time is long, while other biosensors can analyze CRP within 1 min, but they have a relatively high LOD. On the other hand, the proposed self-compensated GMR biosensor provides both a low LOD of 1.95 × 10^−8^ g/mL and a reasonable detection time of 60 min. In addition, the low cost of the developed GMR biosensors and readout system may enable high-throughput industrial mass production. Furthermore, our simple and compact self-compensated GMR biosensing system does not require bulky and costly instruments such as tunable lasers or highly precise spectrometers, thus being ideal for point-of-care diagnostic applications. Its unique advantages render our self-compensated GMR biosensing system suitable for clinical applications such as the rapid diagnosis of sepsis.

## 4. Conclusions and Discussion

We propose and validate a cost-effective, highly stable, intensity-detection-based GMR optofluidic biosensing system for rapid CRP detection. A compensation technique is introduced to suppress the system noise caused by environmental factors, enabling highly stable and real-time analyte detection. From three compensation algorithms, the highest-performing one provided low noise and high sensitivity, substantially improving the RI resolution of the system by up to 180%. Experimental results of CRP detection demonstrate the accurate measurement of the biochemical reaction between CRP and anti-CRP with low noise and long-term stability by reducing the influence of system noise and environmental factors. The system demonstrates an excellent LOD of 1.95 × 10^−8^ g/mL and a reasonable detection time of ~20 min. The low cost, compactness, low noise, long-term stability, and suitable LOD of the biosensor and complete self-compensated GMR biosensing system suggest applicability for point-of-care solutions such as rapid sepsis diagnosis.

## Figures and Tables

**Table 1:** Performance comparison of biosensing system without and with compensation.

Compensation Technique	Noise σ	R^2^	Sn (RIU^−1^)	Resolution (RIU)
No compensation	$1.56\times 10^{-5}$	0.99699	0.181	8.62 × 10^−5^
Direct signal-difference compensation	$9.90\times 10^{-6}$	0.99669	0.190	5.21 × 10 ^−5^
Weighted signal magnification compensation	$8.69\times 10^{-6}$	0.99711	0.239	3.64 × 10^−5^
WDDAM compensation	$5.69\times 10^{-6}$	0.99711	0.186	3.07 × 10^−5^

**Table 2:** Analytic performance of proposed self-compensated GMR optofluidic biosensing system and similar systems.

Biosensor	LOD (g/mL)	Detection time	Reference
SPR biosensor	$3.034\times 10^{-7}$	-	[2]
Fiber-optic biosensor	$6.25\times 10^{-8}$	1 min	[3]
GMR biosensor	$3.2\times 10^{-9}$	2 h	[6]
Amperometric biosensor	$3\times 10^{-10}\;\sim 1\times 10^{-7}$	3 h	[62]
VFA biosensor	$10^{-8}\;\sim \;1\times 10^{-5}$	2 min	[63]
Nanophotonic biosensor	$1.9478\times 10^{-8}$	30 min	[64]
SPR biosensor	$5\times 10^{-9}$	30 min	[65]
MZI biosensor	$10^{-9}$	–	[66]
CVD biosensor	$3.26\times 10^{-10}$	40 min	[67]
POC biosensor	$1.8\times 10^{-5}$	1 min	[68]
LFT biosensor	$3.9\times 10^{-9}$	–	[69]
Self-compensated GMR biosensor	$1.95\times 10^{-8}$	20 min	This study

VFA, vertical flow immunoassay; SPR, surface plasmon resonance; MZI, Mach–Zehnder interferometry; CVD, cardiovascular disease; POC, point-of-care; LFT, lateral flow assay.

## Data Availability

The data presented in this study are available on request from the corresponding author.
